# Cytokine response to lipoprotein lipid loading in human monocyte-derived macrophages

**DOI:** 10.1186/1476-511X-5-17

**Published:** 2006-06-26

**Authors:** Jenny Persson, Jan Nilsson, Marie W Lindholm

**Affiliations:** 1 Clinical Sciences Malmö, CRC House 91:12, UMAS Ing. 72, 20502 Malmö, Sweden

## Abstract

**Background:**

Macrophage foam cell formation is a prominent feature of human atherosclerotic plaques, usually considered to be correlated to uptake of and inflammatory response to oxidized low density lipoproteins (OxLDL). However, there are alternative pathways for formation of macrophage foam cells and the effect of such lipid loading on macrophage function remains to be fully characterized. In the present study we investigated basal and inducible cytokine expression in primary human macrophages either loaded with triglycerides through incubation with very low density lipoproteins (VLDL) or with cholesterol through incubation with aggregated LDL (AgLDL). We then analyzed how foam cell lipid content affected secretion of three pro-inflammatory cytokines: interleukin-1β (IL-1β), interleukin-6 (IL-6) and tumor necrosis factor-α (TNF-α), and of one chemokine: interleukin-8 (IL-8), all of which are considered pro-inflammatory, pro-atherosclerotic, and are expressed by cells in atherosclerotic tissue.

**Results:**

Formation of triglyceride-loaded foam cells resulted in a four-fold increase in basal IL-1β secretion, whereas cholesterol loading lacked significant effect on IL-1β secretion. In contrast, secretion of TNF-α and IL-6 decreased significantly following both cholesterol and triglyceride loading, with a similar trend for secretion of IL-8. Lipid loading did not affect cell viability or expression of caspase-3, and did not significantly affect macrophage ability to respond to stimulation with exogenous TNF-α.

**Conclusion:**

Lipid loading of primary human macrophages resulted in altered cytokine secretion from cells, where effects were similar regardless of neutral lipid composition of cells. The exception was IL-1β, where triglyceride, but not cholesterol, lipid loading resulted in a stimulation of basal secretion of the cytokine. It is apparent that macrophage cytokine secretion is affected by lipid loading by lipoproteins other than OxLDL. As both VLDL and AgLDL have been found in the vessel wall, macrophage cytokine response to uptake of these lipoproteins may have a direct effect on atherosclerotic development *in vivo*. However, macrophage neutral lipid amount and composition did not affect cellular activation by exogenous TNF-α, making it likely that lipoprotein lipid loading can affect foam cell cytokine secretion during basal conditions but that the effects can be overruled by TNF-α during acute inflammation.

## Background

The macrophage foam cell is a prominent feature of all stages of atherosclerosis, but the effect of foam cell transformation on macrophage functional properties remains to be fully characterized. A body of evidence has identified uptake of oxidized low density lipoproteins (OxLDL) as the major cause of foam cell formation. OxLDL contains a number of highly pro-inflammatory and cytotoxic substances making it difficult to differentiate between the direct effects of these components and the effect of lipid loading in itself on macrophage function. However, there are also alternative pathways for formation of macrophage foam cells, including uptake of very low density lipoproteins (VLDL) and aggregated low density lipoproteins (AgLDL). It has been demonstrated that VLDL can penetrate the endothelium [1], and apolipoprotein B-containing lipoprotein particles of VLDL size and density have been isolated from human atherosclerotic tissue [2]. AgLDL can be prepared in vitro by phospholipase treatment of native LDL or by a simple vortex procedure. It is believed that LDL aggregation also occurs in vivo, in particular on the surface of extra cellular matrix components such as chondroitin sulphate. AgLDL has been found in atherosclerotic plaques of apolipoprotein E knock out mice [3] where AgLDL levels positively correlated with severity of plaque development.

We have previously shown that incubation with VLDL enhances macrophage interleukin-1β (IL-1β) expression in a caspase-1 dependent manner [4] and potentiates LPS-induced expression of TNF-α by stimulation of mitogen-activated protein kinase-ERK kinase 1/2 (MEK 1/2) suggesting a pro-inflammatory effect of lipid-loading with triglyceride-rich lipoproteins. In contrast, a reduced expression of tumor necrosis factor-α (TNF-α) has been observed in macrophages incubated with acetylated LDL [5]. These observations suggest that lipid loading may have a direct effect on macrophage functional properties and that these effects may differ depending on lipid composition of the ingested lipoprotein. The aim of the present study was to characterize how macrophage lipid loading affects basal and TNF-α-induced cytokine secretion and if this secretion is influenced by lipid composition of lipid-loaded cells. By incubating primary human macrophages with either AgLDL or VLDL we created a model system for foam cell formation with cholesterol ester or triglycerides as main component of intracellular lipid droplets. We then analyzed how foam cell lipid content affected secretion of three pro-inflammatory cytokines: IL-1β, IL-6 and TNF-α, and of one chemokine: IL-8, all of which are expressed by cells in atherosclerotic plaques [6-9]. IL-1β and TNF-α are involved in macrophage activation, and IL-6 can be induced by both IL-1β and TNF-α and represents secondary response to macrophage activation [10]. IL-8 is a chemoattractant for several cell types present in atherosclerotic tissues, such as T-cells and smooth muscle cells, and has been reported to trigger monocyte adhesion to endothelial cells, an early event in atherosclerosis development [11]. Macrophages isolated from human atherosclerotic plaques release high levels of IL-8, and cholesterol loading has been suggested to trigger macrophage IL-8 production [9]. Our results indicate that both cholesterol loaded and triglyceride loaded cells have decreased basal secretion of pro-inflammatory cytokines, with the exception for IL-1β where basal secretion was stimulated by triglyceride loading. In contrast, lipid loading did not influence the level of cytokine secretion in response to stimulation with exogenous TNF-α.

## Results

### Lipid loading of cells

**Figure 1:**
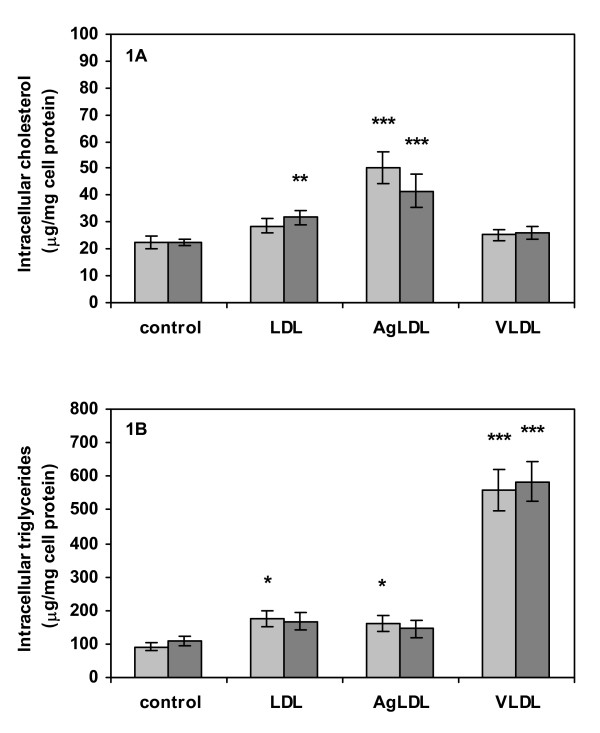
**Intracellular lipid content**. Lipid content of cells after 16 h lipoprotein pre-treatment and 4 h conditioning with or without exogenous TNF-α (1 ng/ml). (A) Intracellular levels of total cholesterol (free cholesterol and cholesterol ester) normalized to cell protein, (B) intracellular triglyceride levels normalized to cell protein. Data represent mean ± SEM of cells from six different blood donors. * = P < 0.05, ** = P < 0.01 and *** = P < 0.001 for comparisons of intracellular lipid content of lipoprotein treated vs. non-treated cells. Light grey = non-stimulated cells, dark grey = cells stimulated by incubation with TNF-α.

Human macrophages isolated from 6 different blood donors were incubated for 16 h in serum-free media with or without addition of 50 μg/ml of native LDL, AgLDL or VLDL. The cells were incubated in lipoprotein-free media for an additional 4 h in order to minimize potential confounding effects of extracellular lipoproteins on subsequent analysis of cytokine secretion. Incubation with AgLDL resulted in a two-fold increase of intracellular cholesterol and a modest increase of intracellular triglyceride levels, while incubation with native LDL only induced a minor increase in intracellular triglycerides (Fig. 1). Macrophages incubated with VLDL demonstrated a six-fold increase in triglycerides, with no significant effect on intracellular cholesterol levels (Fig. 1). The variation in final cholesterol and triglyceride levels between cells from different donors was relatively minor. Activation of cells by addition of 1 ng/ml of TNF-α during the final 4 h of incubation induced a modest increase in total cholesterol levels in cells exposed to native LDL, but was otherwise without effect on intracellular lipid levels (Fig. 1).

### Cytokine secretion

**Figure 2:**
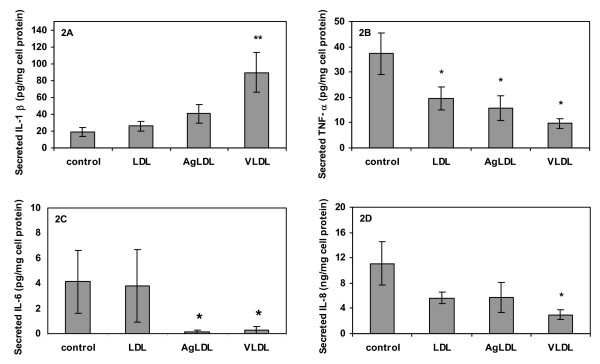
**Cytokine secretion during basal conditions**. Primary human monocytes differentiated for four days were pre-incubated for 16 h serum-free media in presence or absence of LDL, AgLDL or VLDL (50 μg/ml). After pre-incubation, fresh media without lipoproteins, serum or growth factors was conditioned for 4 h and analyzed for cytokine protein content by ELISA. (A) Secreted IL-1β normalized to cell protein, (B) secreted TNF-α normalized to cell protein, (C) secreted IL-6 normalized to cell protein, (D) secreted IL-8 normalized to cell protein. Data represent mean ± SEM of cells from six different blood donors. * = P < 0.05, ** = P < 0.01 for respective lipoprotein treatment compared to control.

**Figure 3:**
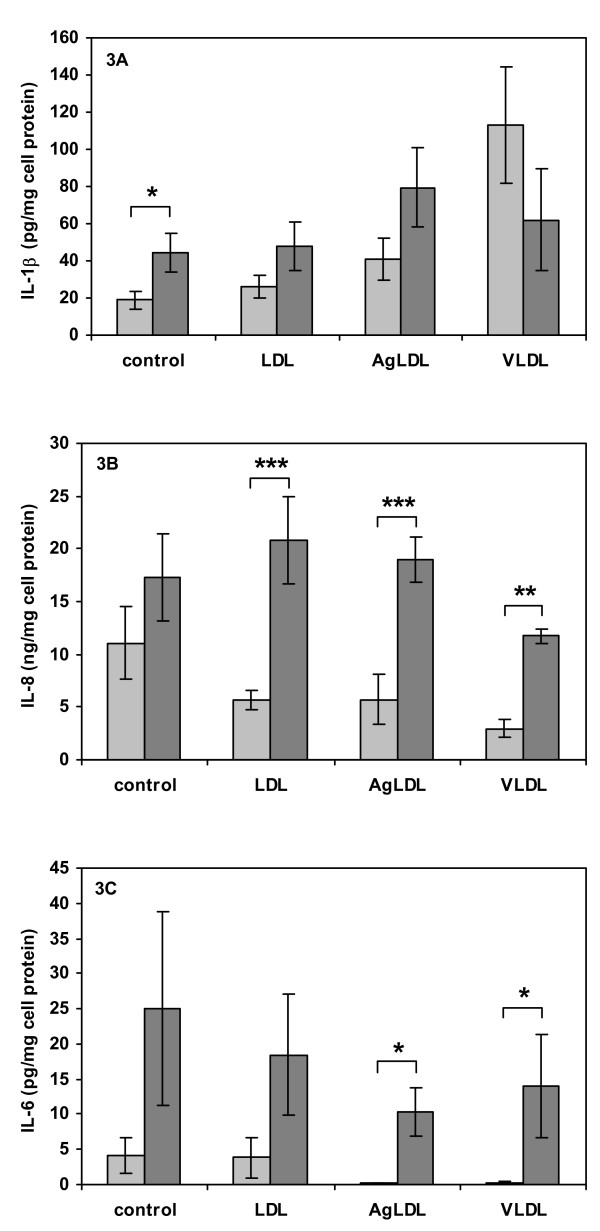
**Cytokine secretion during stimulated conditions**. Cell were pre-treated for 16 h with or without lipoproteins (50 μg/ml), then activated by incubation with exogenous TNF-α (1 ng/ml) for 4 h. For each lipoprotein pre-treatment, significances indicate comparisons between cells incubated in absence or presence of TNF-α. (A) Secreted IL-1β normalized to cell protein, (B) secreted IL-8 normalized to cell protein, (C) secreted IL-6 normalized to cell protein. Data represent mean ± SEM of cells from six different blood donors. * = P < 0.05, ** = P < 0.01 and *** = P < 0.001.

Control macrophage secretion of IL-1β, IL-6 and TNF-α ranged between 4 and 40 pg/mg cell protein, whereas control macrophage secretion of IL-8 was higher by several orders of magnitude (Fig. 2). The variability in cytokine and IL-8 expression between individual donors was higher than that observed for intracellular lipid content. As earlier reported by us [4], formation of triglyceride-loaded foam cells by incubation with VLDL resulted in a significant increase in IL-1β secretion from the cells. In contrast to this, macrophage cholesterol loading by incubation with AgLDL had no significant effect on IL-1β secretion (Fig. 2A). The effect of triglyceride loading on IL-1β secretion differed markedly from that observed for the other two cytokines and IL-8; secretion of TNF-α and IL-6 decreased significantly in both triglyceride and cholesterol loaded cells (Fig 2B and 2C). In addition, IL-8 secretion was significantly decreased by triglyceride loading with VLDL, and a similar trend was observed following cholesterol loading with AgLDL (Fig. 2D). Pre-incubation of macrophages with LDL decreased basal secretion of TNF-α, but had no other significant effect on cytokine and IL-8 secretion (Fig. 2B). Treatment with 1 ng/ml of TNF-α generally stimulated the secretion of IL-1β, IL-6, and IL-8 in control as well as in lipid loaded cells. Cholesterol loading did not affect the macrophage ability to respond to TNF-α stimulation (Fig. 3A–C). Triglyceride-loaded cells responded to activation by exogenous TNF-α with stimulated secretion of IL-6 and IL-8 compared to basal secretion from the same preparations of triglyceride-loaded cells (Fig 3B and 3C), whereas there was no significant difference in IL-1β secretion from triglyceride-loaded cells incubated in absence or presence of exogenous TNF-α (Fig 3A).

### Cell viability and apoptosis

Cell viability was controlled by the MTT assay and by analysis of LDH leakage to incubation media. No treatment induced decreased cell viability as monitored by these two methods (data not shown). However, it is possible that the observed cytokine levels in media conditioned with lipid loaded cells are correlated to apoptotic responses not noticeable by cell viability assays. As our previous data demonstrated a connection between IL-1β secretion and a marker for apoptotic development, we used two independent parameters, caspase-3 activity and quantification of DNA laddering, to evaluate apoptosis in the current experiments. Neither apoptotic parameter was significantly altered in cells treated with native or aggregated lipoproteins, whereas oxidized LDL used as positive control gave a strong signal with both methods for apoptosis evaluation (data not shown).

## Discussion

The present results demonstrate that lipid loading had an inhibitory effect on basal macrophage secretion of some, but not all, analyzed pro-inflammatory cytokines, whereas lipid loading did not influence the macrophage ability to respond to stimulation with exogenous stimuli, such as TNF-α. Both triglyceride and cholesterol lipid loading resulted in significantly decreased secretion of the pro-inflammatory cytokines TNF-α and IL-6 compared to control cells incubated in absence of lipoproteins. There was also a trend towards decreased secretion of IL-8. However, the effect of lipid loading on secretion of IL-1β was markedly different. Triglyceride loading by incubation with VLDL was associated with a strong stimulation of basal IL-1β secretion. It is unlikely that cytokines recovered from conditioned media are the result of cytotoxic effects of either lipoprotein treatment. First, lipoproteins were controlled for endotoxins and oxidation products before used in experiments. Second, neither lipoprotein lipid loading treatment induced cell death, as judged by the MTT assay and by LDH leakage to conditioned media compared to controls.

It has previously been shown that the increased IL-1β secretion from macrophages exposed to VLDL in part can be explained by stimulation of post-translational processes involving activation of caspase-1, the enzyme that cleaves the IL-1β pro-peptide prior to exocytosis. Expression of IL-1β and TNF-α are both regulated by the transcription factor NF-κB, and in endothelial cells NF-κB activity is up-regulated by triglyceride loading induced by VLDL [12], making up-regulation of NF-κB activity a likely candidate for the observed effect on IL-1β secretion from triglyceride loaded cells but an unlikely candidate for regulation of the other analyzed cyto- and chemokines. It is also possible that some of the observed effects of lipid loading were mediated by peroxisome proliferators activated receptor-γ (PPAR-γ) as this nuclear receptor has been reported to be up-regulated by oxidized LDL [13] and PPAR-γ has been reported to negatively regulate expression of IL-1β, IL-6 and TNF-α [14]. However, during basal conditions there was no positive correlation between IL-1β and TNF-α secretion neither in triglyceride-rich nor in cholesterol-rich cells. In fact, secretion of the two cytokines appeared to be inversely regulated suggesting involvement of additional regulatory mechanisms. Pre-incubation of macrophages with LDL significantly lowered basal secretion of TNF-α but had no significant effect on macrophage secretion of IL-1β, IL-6, or IL-8, indicating that either intracellular lipid level needs to reach a threshold level or signaling events not mediated through the LDL receptor are required for the panel of effects on macrophage cytokine secretion that appears to be induced by triglyceride or cholesterol loading. A full explanation to why macrophage triglyceride lipid loading by VLDL uptake, but not cholesterol lipid loading by uptake of AgLDL, results in opposing effects on IL-1β vs. IL-6, IL-8 and TNF-α secretion still remains to be elucidated, but it is possible that the earlier reported stimulation of IL-1β release by VLDL stimulation of caspase-1 activity is responsible for this difference.

The observation that macrophage triglyceride loading by VLDL but not cholesterol loading by AgLDL stimulates expression of IL-1β is of interest against the background of epidemiological findings of a strong association between VLDL and inflammatory makers such as C-reactive protein (CRP), while no, or only weak, such associations are found between LDL and inflammatory makers. Hypertriglyceridemia is prevalent in diabetic patients, whereas hypercholesterolemia is associated both with an increased risk for LDL oxidation and a high risk for formation of aggregated LDL in the intimal matrix of the vascular wall. Oxidized LDL exerts strong effects on macrophage cytokine signaling – effect usually pinpointed to specific oxysterols and other lipid oxidation products – and therefore the main focus for studies of lipoprotein effects on macrophage foam cell inflammatory response has been on oxidized lipoproteins and specific lipid species. Our present data illustrates that human macrophages respond to lipid loading induced by non-oxidized lipoproteins with significant alterations in basal cytokine secretion, an effect most pronounced in triglyceride-rich but also induced in cholesterol-rich cells. It is apparent from our study and others [12] that lipoprotein oxidation is not a prerequisite for lipoprotein influence on macrophage immune response. Support for a direct effect of lipid-loading on macrophage function has also come from animal studies demonstrating decreased expression of the growth factor PDGF-B in atherosclerotic plaque foam cells as compared to macrophages with no visible lipids [15].

## Conclusion

In conclusion, lipid loading of primary human macrophages results in altered cytokine secretion from cells, where effects were similar regardless of neutral lipid composition of cells. The exception was IL-1β, where triglyceride, but not cholesterol, lipid loading resulted in a stimulation of basal secretion of the cytokine. However, as exogenous TNF-α did activate both lipid loaded and control cells regardless of foam cell neutral lipid composition, and tissue levels of TNF-α are relatively high in atherosclerotic plaques, potentially anti-inflammatory effects of neutral lipid loading are likely to be of minor importance in atherosclerotic tissue during active inflammatory response.

## Methods

### Lipoprotein preparation

Lipoproteins were prepared by differential ultra centrifugation of fresh plasma from healthy donors [16]. Lipoproteins were stored at -80°C with 10% sucrose and 4.8 mM EDTA, pH = 7.4, a procedure which does not affect lipoprotein integrity [17]. Lipoproteins were thawed and desalted immediately before use and protein content determined with BCA protein assay reagent (Pierce). Lipoprotein oxidation was determined by the conjugated diene assay [18]. Lipoprotein fraction integrity was confirmed by agarose gel electrophoresis (Sebia, France). Endotoxin content of lipoprotein preparations were <0.03 EU/ml as determined by the Limulus assay (E-toxate, Sigma). Aggregated LDL was prepared by vortexing LDL preparations for 3 min just before use [19]. Oxidized LDL was prepared by incubation of LDL with 5 μM CuSO_4 _at 37°C for 48 h.

### Cell culture

Primary human monocytes were isolated from buffy coats from healthy donors [20] and seeded in 6-well plates at a cell density of 2 × 10^6 ^cells/well. Cells were maintained for 4 × 24 hours in basal media: Macrophage-serum free media (Macrophage-SFM) with transferrin (25 μg/ml), glutamine (2 mM), gentamicin (50 μg/ml), and granulocyte macrophage-colony stimulating factor (GM-CSF, 10 ng/ml), at 37°C with 5% CO_2_. After this, media was changed to basal media without GM-CSF for 24 h, with or without lipoproteins (50 μg/ml) present for the last 16 h. Cytotoxic effects of lipoprotein treatments was assessed by the MTT (methylthiazoletetrazolium) assay (Sigma). Finally, cells were rinsed and fresh GM-CSF and lipoprotein free media with or without exogenous TNF-α (final concentration 1 ng/ml) was conditioned with treated cells for 4 hours. Cell viability was assessed by analyzing leakage of LDH (lactate dehydrogenase assay, Sigma). Conditioned media were removed, protease inhibitor was added (PMSF, final concentration 1 mM) and media were centrifuged at 1000*g *for 5 minutes to remove floating cells or cell debris.

### Chemical analyses

Cell lipids were extracted with hexane:isopropanol (3:2 + 0.005% butylhydroxytoluene), dried down under N_2 _and reconstituted in ethanol. Lipid analyses (triglycerides and total cholesterol) were performed with colorimetric assays (Sigma). After lipid extraction, residual cell proteins were dissolved in 0.3 M NaOH and cell lysates used for cell protein analyses with BCA protein reagent (Pierce).

### Cytokine analyses

Interleukin-8 (IL-8) and interleukin-1β (IL-1β) were analyzed by commercial ELISA sets (R&D systems). Interleukin-6 (IL-6) and tumor necrosis factor-α (TNF-α) were analyzed with matched antibodies (Dako, Denmark) in high-sensitivity sandwich ELISAs developed in the laboratory [21].

### Apoptosis assessment

Apoptosis was evaluated using two independent methods. Cells incubated with OxLDL were used as positive controls. Cell lysates after lipoprotein treatment were analyzed for caspase-3 activity according to manufacturer's instructions (BD Biosciences). For DNA laddering analysis, cell homogenates in PBS were separated in cytosolic and nuclear fractions, after which DNA was released from histones by addition of 5 M NaCl before precipitation with isopropanol. DNA was rinsed with ice cold ethanol, pelleted by centrifugation at 20 000*g *and pellets dissolved in TE buffer (1 mM EDTA, 10 mM Tris-HCl, pH = 7.4) before being separated on agarose gels.

### Statistical analyses

Experiments were performed using cells isolated from six different donors, with analyses performed in duplicates for each donor. All data are reported as mean ± SEM. Statistical differences are calculated using Mann-Whitney non-parametric test.

## Competing interests

The author(s) declare that they have no competing interests.

## Authors' contributions

JP participated in cell culture, lipoprotein characterization and ELISA assays. JN participated in design and coordination of the study. MWL designed the study, carried out most of the experiments and drafted the manuscript. All authors read and approved the final manuscript.
